# Identification of a novel mutation of NOG in family with proximal symphalangism and early genetic counseling

**DOI:** 10.1186/s12881-019-0917-5

**Published:** 2019-11-06

**Authors:** Cong Ma, Lv Liu, Fang-Na Wang, Hai-Shen Tian, Yan Luo, Rong Yu, Liang-Liang Fan, Ya-Li Li

**Affiliations:** 1 Departments of Reproductive Genetics, HeBei General Hospital, ShiJiaZhuang, 050051 China; 2 Department of Respiratory Medicine, Diagnosis and Treatment Center of Respiratory Disease, Diagnosis and Treatment Center of Respiratory Disease, the Second Xiangya Hospital of Central South University, Changsha, 410011 Hunan China; 3 Departments of Anesthesiology, the Second Xiangya Hospital, Central South University, Changsha, 410011 China; 4 Department of Cell Biology, The School of Life Sciences, Central South University, Changsha, 410011 Hunan China

**Keywords:** Proximal symphalangism, Deafness, NOG mutation, Prenatal diagnosis

## Abstract

**Background:**

Proximal symphalangism is a rare disease with multiple phenotypes including reduced proximal interphalangeal joint space, symphalangism of the 4th and/or 5th finger, as well as hearing loss. At present, at least two types of proximal symphalangism have been identified in the clinic. One is proximal symphalangism-1A (SYM1A), which is caused by genetic variants in *Noggin* (*NOG*), another is proximal symphalangism-1B (SYM1B), which is resulted from *Growth Differentiation Factor 5* (*GDF5*) mutations.

**Case presentation:**

Here, we reported a Chinese family with symphalangism of the 4th and/or 5th finger and moderate deafness. The proband was a 13-year-old girl with normal intelligence but symphalangism of the 4th finger in the left hand and moderate deafness. Hearing testing and inner ear CT scan suggested that the proband suffered from structural deafness. Family history investigation found that her father (II-3) and grandmother (I-2) also suffered from hearing loss and symphalangism. Target sequencing identified a novel heterozygous *NOG* mutation, c.690C > G/p.C230W, which was the genetic lesion of the affected family. Bioinformatics analysis and public databases filtering further confirmed the pathogenicity of the novel mutation. Furthermore, we assisted the family to deliver a baby girl who did not carry the mutation by genetic counseling and prenatal diagnosis using amniotic fluid DNA sequencing.

**Conclusion:**

In this study, we identified a novel *NOG* mutation (c.690C > G/p.C230W) by target sequencing and helped the family to deliver a baby who did not carry the mutation. Our study expanded the spectrum of *NOG* mutations and contributed to genetic diagnosis and counseling of families with SYM1A.

## Background

Proximal symphalangism is a rare genetic disorder of congenital limb malformation, characterized by ankylosis of the proximal interphalangeal joints, carpal and tarsal bone fusion, and, in some cases, conductive deafness and premature ovarian failure [1, 2]. The typical features of proximal symphalangism are reduced proximal interphalangeal joint space, symphalangism of the 4th and/or 5th finger [3, 4]. As early as in 1916, Cushing has described an American family with ankylosis of the proximal interphalangeal joints, and he named this heterozygote autosomal dominant disease as symphalangism [5].

At present, there are two types of diseases in the proximal symphalangism family: (1) Proximal symphalangism-1A (SYM1A, OMIM # 185800), which iss caused by genetic variants in *Noggin* (*NOG*) [6, 7]; (2) Proximal symphalangism-1B (SYM1B), which is resulted from *Growth Differentiation Factor 5* (*GDF5*) mutations [8, 9]. In addition, some other diseases may be also related to proximal symphalangism, such as tarsal-carpal coalition syndrome, multiple synostoses syndrome, and brachydactyly, etc. [10, 11].

In this study, we employed target sequencing to explore the genetic lesion of a Chinese family with symphalangism of the 4th and/or 5th finger and moderate deafness. A novel mutation (c.690C > G/p.C230W) of *NOG* was identified in all affected individuals in this family. Furthermore, after genetic counseling and prenatal diagnosis with us, the mother successfully delivered a baby girl who did not carry the mutation.

## Case presentation

**Fig. 1:**
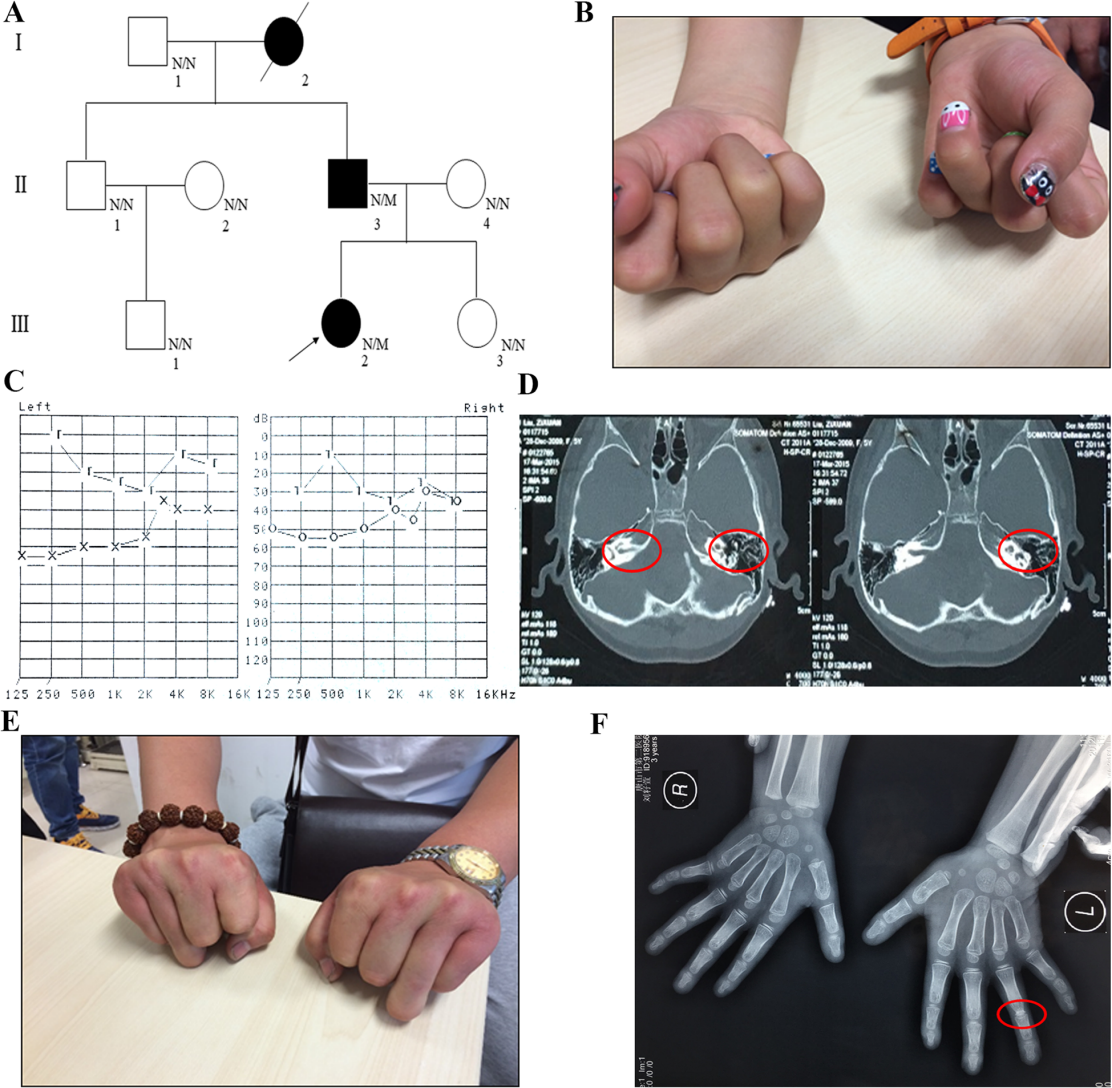
The Clinical data of the family. **a** The pedigree of this family. Black circles/squares are affected, white circles/squares are unaffected. N means Normal, M indicates Mutation. Arrow indicates the proband. **b** The proband showed the symphalangism of the 4th finger in the left hand. **c** Hearing testing suggests the proband suffering from moderate deafness. **d** Inner ear CT showed abnormal inner ear structure and abnormal calcification. The red circles marked the abnormal regions which indicated cochlear hypoplasia, inner ear bone thickening and increased density. **e** The symphalangism of the 4th finger in II-3. **f** Hands X-ray of III-2. The red circles marked the abnormal regions

A family from North of China (Hebei Province) with eight members across three generations participated in the study (Fig. 1a). The proband (III-2) was a 13-year-old girl with normal intelligence but symphalangism of the 4th finger in the left hand (Fig. 1b) and moderate deafness (Fig. 1c). Inner ear CT scan found abnormal inner ear structure (cochlear hypoplasia) and abnormal calcification (inner ear bone thickening and increased density) (Fig. 1d). Family history investigation found that her father (II-3) and grandmother (I-2) also suffered from hearing loss and symphalangism (Fig. 1a, e). Her grandmother has died six years ago. Her father showed the symphalangism of the 4th finger in left hand (Fig. 1e, f). He had performed the vestibulotomy and recovered the hearing one year ago. They went to the Department of Reproductive Genetics, HeBei General Hospital because the mother was pregnant with the second baby. They wanted to detect whether the second baby was normal or not.

### Genetic analysis

**Fig. 2:**
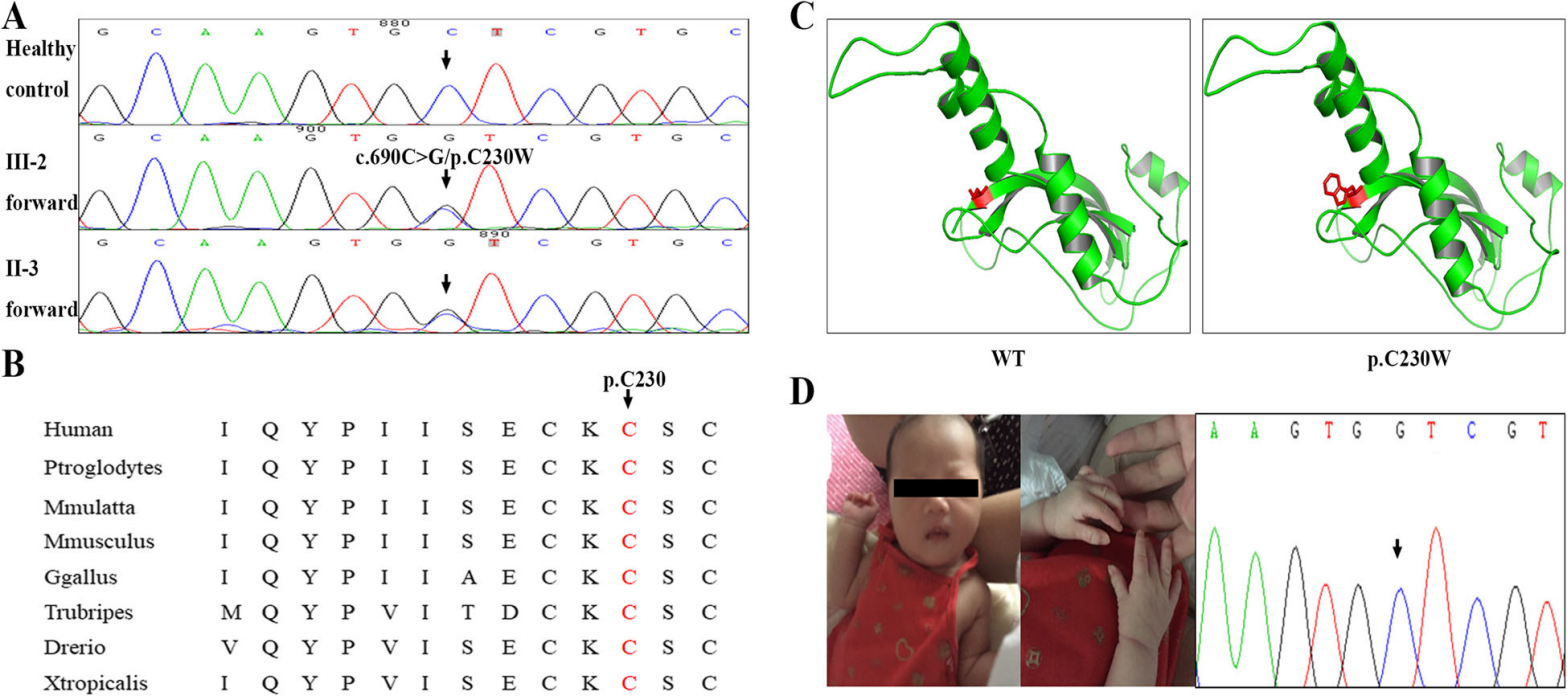
Genetic analysis of the family. **a** Sanger DNA sequencing chromatogram demonstrates the heterozygosity for a *NOG* mutation (c.690C > G/p.C230W). **b** Analysis of the mutation and protein domains of Noggin. The C230 affected amino acid locates in the highly conserved amino acid region in different mammals (from Ensembl). The black arrow and red words show the C230 site. **c** Swiss-model analyzed the Noggin structures of WT and Mutated (p.C230W). **d** The healthy hands of III-3 and normal sequences of amniotic fluid DNA

We selected the proband’s genomic DNA to perform the target sequencing to detect the disease-causing mutations by Sinopath Diagnosis Company (Beijing, China). Target sequencing yielded 3.71 Gb of data with 99.088% coverage of the target region and 97.530% of the target covered over 10×. After filtering dbSNP132, 1000G, EXAC, and GenomAD database (MAF < 0.01), only 12 mutation were left. We then conducted the co-segregation analysis by Sanger sequencing and only seven variants were exist in affected individuals and were absent in healthy members (Table 1). We further performed the bioinformatics analysis including MutationTaster, SIFT, Polyphen-2, PANTHER, ToppGene function analysis, OMIM clinical phenotype analysis and ACMG classification (Table 1), we highly suspected the novel mutation (c.690C > G/p.C230W) of *NOG,* belonging to PM1 and PM2 in ACMG guidelines [12], was responsible for the family with SMY1A. This mutation resulted in a substitution of in polar amino acid cysteine by nonpolar amino acid tryptophan in the codon 230 of exon 1 of *NOG* gene, and was not presented in our 200 control cohorts. Noggin amino acid sequence alignment analysis suggested that this mutation was located in a highly evolutionarily conserved site (Fig. 2b). In addition, we also constructed a part model of the Noggin protein using SWISS-MODEL (https://swissmodel.expasy.org) (Fig. 2c) and, after applying SDM software (http://marid.bioc.cam.ac.uk/sdm2/prediction) to analyze the structure, it was found that this novel mutation might increase the solvent accessibility (WT:16.9% and Mutant: 39.9%) and reduce the stability of the Noggin protein.

**Table 1:** The mutations list after data filtering and co-segregation analysis

CHR	POS	RB	AB	Gene	Mutation	SIFT	PolyPhen-2	MutationTaster	PANTHER	OMIM clinical phenotype	ToppGene function	ACMG classification
1	45,481,060	C	T	*UROD*	NM_000374: c.994C > T, p.R332C	0,D	0.94,D	0.99,D	–	AD or AR: Porphyria cutanea tarda	heme biosynthetic process	BP5
2	149,216,410	G	A	*MBD5*	NM_018328: c.83G > A, p.R28H	0,D	0.99,D	0.99,D	P	AD: Mental retardation	response to growth hormone	BP5
2	189,953,479	G	T	*COL5A2*	NM_000393: c.587G > T, p.A196D	0.29,T	0.98,D	0.99,D	–	AD: Ehlers-Danlos syndrome	regulation of endodermal cell differentiation	BP4, BP5
3	38,674,642	G	A	*SCN5A*	NM_198056: 157G > A, p.R53W	0,D	0.36,B	0.95,D	P	AD: Atrial fibrillation	voltage-gated sodium channel activity	BP4, BP5
3	184,953,112	G	A	*EHHADH*	NM_001966: c.317G > A, p.A106V	0,D	0.99,D	0.99,D	P	AD: Fanconi renotubular syndrome	peroxisomal transport	BP5
17	48,701,856	G	A	CACNA1G	NM_018896: c.6365G > A, p.R2122H	0.04,D	0.01,B	0.8,D	P	AD: Spinocerebellar ataxia	voltage-gated calcium channel	BP4, BP5
17	54,672,274	C	G	*NOG*	NM_005450: c.690C > G, p.C230W	0,D	0.99,D	0.99,D	D	AD: Symphalangism proximal	fibroblast growth factor receptor signaling pathway	PM1, PM2

*CHR* Chromosome, *POS* position, *RB* reference sequence base, *AB* alternative base identified, *D* damaging, *P* probably damaging, *B* Benign, *T* Tolerated, *AR* autosomal recessive, *AD* autosomal dominant, *BP* Benign Supporting, *PM* Pathogenicity Moderate

### Prenatal diagnosis

When the parents came to our hospital, the mother has been pregnant with the second baby for 17 weeks and they wanted to have a healthy baby. According to ACMG classification, the novel mutation (c.690C > G/p.C230W) of *NOG* belongs to PM1 and PM2. Simultaneously, target sequencing only identified this mutation as a pathogenic variant. So, we highly believed the novel mutation (c.690C > G/p.C230W) of *NOG* was the genetic lesion of the family with proximal symphalangism and hearing loss. We then performed the Sanger sequencing of amniotic fluid DNA to detect the mutation, fortunately, the results showed a normal allele of the second baby. And 22 weeks later, the mother delivered a 3.4-kg healthy girl (Fig. 2d).

## Discussion

The human *NOG* gene encoding Noggin protein is located on chromosome 17q22, and it consists of one exon, spanning approximately 1.9 kilobases (kb) [6]. Noggin protein is involved in the development of many body tissues, including nerve tissue, muscles, and bones and the role of Noggin in bone development makes it significant for proper joint formation [13]. According to previous researches, Noggin protein can interact with bone morphogenetic proteins (BMPs) and regulate the development of bone and other tissues [14]. In detail, the Noggin protein regulates the activity of BMPs by binding to them and blocking them from attaching to the downstream receptor, which results in a decrease in BMP signaling [15]. In our research, the novel mutation (c.690C > G/p.C230W) of *NOG* can increase the solvent accessibility and reduce the stability of the Noggin, which may active the BMP signal pathway and lead to bone diseases.

In 1999, five *NOG* mutations were identified in unrelated families with symphalangism (SYM1A) and a de novo mutation in a patient with unaffected parents [6]. Interestingly, a wide variety of bone development anomalies, including tarsal/carpal coalition syndrome [10], brachydactyly [16], multiple synostoses syndrome [17], Stapes ankylosis with broad thumbs and toes [18], have been reported in patients with *NOG* mutations. Similar observations were also reported in the families even with the same mutation [16, 19]. Therefore, the pleiotropic types of bone diseases and significant genetic heterogeneity make it difficult to be diagnosed. We summarized the previous reports and found that approximately 57 mutations (60 patients) of *NOG* have been identified in different types of disorders (Table 2).

**Table 2:** The summary of reported mutations of NOG

No.	Mutation	Phenotypes	Reference
**1**	**p. Leu20fs**	**Multiple synostoses syndrome**	**Takahashi et al. (2001)**
2	p. Pro35Ala	Brachydactyly type B	Lehmann et al. (2007)
**3**	**p. Pro35Ser**	**Teunissen-Cremers syndrome**	**Hirshoren et al. (2008)**
**4**	**p. Pro35Ser**	**Proximal symphalangism**	**Mangino et al. (2002)**
5	p. Pro35Ser	Brachydactyly type B	Lehmann et al. (2007)
6	p. Pro35Arg	Proximal symphalangism	Gong et al. (1999)
7	p. Pro35Arg	Tarsal–carpal coalition syndrome	Dixon et at. (2001)
8	p. Ala36Pro	Brachydactyly type B	Lehmann et al. (2007)
**9**	**p. Pro37Arg**	**Tarsal–carpal coalition syndrome**	**Debeer et al. (2004)**
10	p. Pro42Ala	Multiple synostoses syndrome	Debeer et al. (2005)
11	p. Pro42Ser	Proximal symphalangism	Sha et al. (2019)
12	p. Pro42Arg	Multiple synostoses syndrome	Oxley et al. (2008)
13	p. Pro42Thr	Multiple synostoses syndrome	Aydin, H et al. (2013)
**14**	**p. Val44fs**	**Teunissen-Cremers syndrome**	**Weekamp et al. (2005)**
15	p. Glu48Lys	Brachydactyly type B	Lehmann et al. (2007)
**16**	**p. Glu48Lys**	**Proximal symphalangism**	**Kosaki et al. (2004)**
17	p. Pro50Arg	Tarsal–carpal coalition syndrome	Debeer et al. (2005)
18	p. Asp55Tyr	Proximal symphalangism	Xiong et al. (2019)
**19**	**p. Glu85fs**	**Stapes ankylosis with broad thumb and toes**	**Brown et al. (2002)**
20	p. Arg87fs	Multiple synostoses syndrome	Lee et al. (2014)
21	p. Gly91Cys	Fibrodysplasia ossificans progressiva	Kaplan et al. (2008)
22	p. Gly92Arg	Fibrodysplasia ossificans progressiva	Kaplan et al. (2008)
23	p. Gly92Glu	Fibrodysplasia ossificans progressiva	Kaplan et al. (2008)
24	p. Ala95Thr	Fibrodysplasia ossificans progressiva	Kaplan et al. (2008)
**25**	**p. Ala102fs**	**Proximal symphalangism**	**Thomeer et al. (2011)**
**26**	**p. Gln110X**	**Stapes ankylosis with broad thumb and toes**	**Brown et al. (2002)**
**27**	**p. Leu129X**	**Proximal symphalangism**	**Takahashi et al. (2001)**
**28**	**p.Gln131X**	**Stapes ankylosis with broad thumbs and toes**	**Takashi etal. (2014)**
**29**	**p. Lys133X**	**Stapes ankylosis with broad thumb and toes**	**Takano et al. (2016)**
**30**	**p. Arg136Cys**	**Proximal symphalangism**	**Masuda et al. (2014)**
**31**	**p. Trp150Cys**	**Proximal symphalangism**	**Pan et al. (2015)**
**32**	**p. Cys155Phe**	**Stapes ankylosis with symphalangism**	**Usami et al. (2012)**
33	p. Cys155Ser	Proximal symphalangism	Usami et al. (2012)
34	p. Arg167Gly	Brachydactyly type B	Lehmann et al. (2007)
35	p. Arg167Cys	Proximal symphalangism	Liu et al. (2015)
36	p. Cys184Tyr	Proximal symphalangism	Takahashi et al. 2001
**37**	**p. Cys184Phe**	**Proximal symphalangism**	**Usami et al. 2012**
38	p. Pro187Ser	Brachydactyly type B	Lehmann et al. (2007)
**39**	**p. Pro187Ala**	**Proximal symphalangism**	**Ganaha et al. (2015)**
**40**	**p. Glu188fs**	**Teunissen-Cremers syndrome**	**Weekamp et al. (2005)**
41	p. Gly189Cys	Proximal symphalangism	Gong et al. (1999)
**42**	**p. Met190Val**	**Multiple synostoses syndrome**	**Oxley et al. (2008)**
**43**	**p. Leu203Pro**	**Teunissen-Cremers syndrome**	**Weekamp et al. (2005)**
**44**	**p. Arg204Leu**	**Tarsal/carpal coalition syndrome**	**Dixon et al. (2001)**
45	p. Arg204Gln	Tarsal-carpal coalition syndrome	Das et al. (2018)
46	p. Trp205X	Multiple synostoses syndrome	Dawson et al. (2006)
**47**	**p. Trp205Cys**	**Facioaudiosymphalangism syndrome**	**van den Ende et al. (2005)**
48	p. Trp205fs	Stapes ankylosis with broad thumb and toes	Emery et al. (2009)
**49**	**p. Cys215X**	**Stapes ankylosis with broad thumb and toes**	**Usami et al. (2012)**
50	p. Trp217Gly	Multiple synostoses syndrome	Gong et al. (1999)
**51**	**p. Ile220Asn**	**Proximal symphalangism**	**Gong et al. (1999)**
**52**	**p. Ile220fs**	**Proximal symphalangism**	**Gong et al. (1999)**
53	p. Tyr222Asp	Proximal symphalangism	Gong et al. (1999)
54	p. Tyr222Cys	Proximal symphalangism	Gong et al. (1999)
55	p. Tyr222Cys	Tarsal–carpal coalition syndrome	Dixon et al. (2001)
56	p. Pro223Leu	Proximal symphalangism	Gong et al. (1999)
**57**	**p. Cys228Gly**	**Stapes ankylosis with broad thumb and toes**	**Ishino et al. (2015)**
**58**	**p. Cys228Ala**	**Multiple synostoses syndrome**	**Ganaha et al. (2015)**
**59**	**p. Cys230Tyr**	**Multiple synostoses syndrome**	**Bayat et al. (2016)**
**60**	**p. Cys230Trp**	**Proximal symphalangism**	**Present study**
**61**	**p. Cys232Trp**	**Multiple synostoses syndrome**	**Rudnik-Schöneborn et al. (2010)**

Bold words indicate the patients with deafness

In this study, a family with symphalangism and moderate deafness was investigated by target sequencing. Genetic analysis found a novel mutation (c.690C > G/p.C230W) of *NOG* in two affected members. Of note, both of two patients with p.C230W in the family were associated with hearing loss. To date, 29 mutations have been reported in symphalangism patients related to deafness (Table 2) [11]. And the mutation p.C230W was the fifth report related to *NOG* mutation, although some Chinese journals have also published some reported mutations. Meanwhile, this difference also suggested that there were still a lot of novel mutations need to discovery in Chinese population.

The p.C230W mutation disrupts the cysteine knot motif of the C-terminal domain of Noggin (amino acids 155–232), which contains a series of nine cysteine residues and was shown to target the molecule to a specific receptor protein [20, 21]. The similar mutations (p.C228G, p.C228S, p.C230Y and p.C232W) have been identified in patients with symphalangism and hearing loss, which indicated that mutations in cysteine residues may be related to abnormal development of auditory ossicles and hearing loss [19, 22–24].

In clinical genetics, the aim of mutation detection is to make contributions to genetic diagnosis and counseling. In this study, we identified the genetic lesion of the family by target sequencing. All the filtered data were shown in Table 1. We not only performed the informatics analysis of the novel mutation by multi-different algorithm based bioinformatics programs, but also followed the ACMG guidelines to estimate the pathogenicity of the novel mutation strictly (PM1 and PM2). Finally, we highly believed that the novel mutation (p.C230W) of *NOG* may be the genetic lesion of the family. We then assisted the family to get a healthy baby by amniotic fluid DNA sequencing referring to other people’s research [25]. Prenatal diagnosis not only helped the patient to delivery healthy baby and improved the population quality but also relieved psychological and financial stress [26]. Our study provided a successful example for genetic counseling and prenatal diagnosis of patients with SYM1A.

## Conclusions

We reported a novel *NOG* mutation (c.690C > G/p.C230W) in a three-generation family with SYM1A. And we helped them delivery a girl baby who did not carry the mutation by genetic counseling and prenatal diagnosis. Our study not only presented the important role of *NOG* in proximal symphalangism and deafness but also expanded the spectrum of *NOG* mutations and contributed to genetic diagnosis and counseling of families with SYM1A.

## Data Availability

The datasets used and/or analyzed during the current study are available from the corresponding author on reasonable request.

## Abbreviations

BMPs: Bone morphogenetic proteins
GDF5: Growth Differentiation Factor 5
kb: Kilobases
NOG: Noggin
SYM1A: proximal symphalangism-1A
SYM1B: proximal symphalangism-1B
